# Effects of repeated cigarette smoke extract exposure over one month on human bronchial epithelial organotypic culture

**DOI:** 10.1016/j.toxrep.2018.08.015

**Published:** 2018-08-18

**Authors:** Shigeaki Ito, Kanae Ishimori, Shinkichi Ishikawa

**Affiliations:** Scientific Product Assessment Center, R&D Group, Japan Tobacco Inc., 6-2, Umegaoka, Aoba-ku, Yokohama, Kanagawa, 227-8512, Japan

**Keywords:** CS, cigarette smoke, COPD, chronic obstructive pulmonary disease, GRO, growth factor related oncogene, IL, interleukin, IP-10, interferon gamma-induced protein-10, MCP-1, monocyte chemotactic protein-1, MIP-1β, macrophage inflammatory protein-1β, MMP, metalloproteinase, RANTES, regulated on activation normal T cell expressed and secreted, SDF-1α, stromal cell-derived factor-1α, Cigarette smoke extract, Repeated exposure, Organotypic culture

## Abstract

Cigarette smoke is a known risk factor for inflammatory diseases in the respiratory tract, and inflammatory exacerbation is considered pivotal to the pathogenesis of these diseases. Here, we performed two repeated exposure studies in which we exposed human bronchial epithelial tissues in an organotypic culture model to cigarette smoke extract (CSE); the first study was conducted over a four-day period to determine the suitable dose range for the extended exposure period, and the second was a one-month exposure study to elucidate the exposure-by-exposure effects in bronchial tissues. We focused on matrix metalloproteinase (MMP)-9 and -1/3 and the inflammatory cytokines interleukin (IL)-8 and growth factor related oncogene to evaluate the transition into an inflammatory state. Even at CSE doses with no or low toxicity for a single exposure, the repetition of exposure induced cumulative effects on both the inflammatory responses, specifically the IL-8 and MMPs levels, and tissue morphology. Interestingly, untreated controls initially had relatively high baseline levels of these secreted proteins; these levels gradually declined, after which they showed periodic level changes, suggesting an acclimation period may be needed for this system. These results demonstrate the usability of this system for the elucidation of sub-chronic effects *in vitro*.

## 1 Introduction

Cigarette smoke (CS) is a major risk factor for airway diseases, such as chronic obstructive disease (COPD) [1,2]. In the pathogenesis of such diseases, cells and tissues in the respiratory tract directly interact with the reactive substances in CS, which elicits responses that lead to inflammation. To reproduce and investigate the pathogenesis of inflammatory airway diseases, many *in vivo* studies have been conducted with rodent models, but their results leave some uncertainties about what effects may be due to interspecies differences [3]. Meanwhile, *in vitro* models of respiratory tract tissue have improved over the past decades, and these enhanced models now enable investigations of the effects of test substances to be conducted using human cells that resemble human *in vivo* tissues. The model tissues consist of functionally differentiated ciliated cells, basal cells, and club cells, and these cells form a pseudostratified columnar epithelium [4]. Previous studies showed the similarity between this *in vitro* model and *in vivo* epithelia, in terms of their morphologies and transcriptomes. Pezzulo et al. demonstrated that transcriptional profiles of organotypic culture of bronchial epithelium are comparable with those of airway epithelia *in vivo* [5], and Mathis et al. also reported that such organotypic bronchial epithelia show a similar transcriptional perturbation following exposure to CS compared with human lungs that have inhaled CS [6].

The organotypic culture model has the additional advantage of having a long-shelf life. Baxter et al. demonstrated that, over a few months, organotypic cultured tissues retain their phenotype, including not only morphology but also expression of xenobiotic metabolizing genes and key mucociliary protein markers [7]. Furthermore, Anderson et al. previously conducted work on repeated exposure to limonene and its reaction products using organotypic cultured tissues, and they found increases of some inflammatory cytokines that were not observed following a single exposure in cell lines [8]. These results suggest that organotypic culture models are useful tools for the elucidation of sub-chronic effects.

Only a few studies attempting to elucidate the effects of repeated exposure to CS have been reported. Recently, our group found that over the course of 7 repeated whole CS exposures in organotypic cultured bronchial tissues; inflammatory responses were augmented as exposure repetition increased [9]. We hypothesized that the augmentation could be replicated by exposure to non-toxic dose of cigarette smoke extract (CSE) containing smoke constituents other than those in the gas and vapor phase, and here, to determine suitable repeated exposure conditions of cigarette smoke extract (CSE), we first performed four-day exposures to CSE at intervals of 24 h. We then performed a one-month CSE exposure study with three exposures per week and analyzed the alterations of several inflammatory mediators. For comparative purposes, we also observed the spontaneous inflammatory responses in organotypic culture. To our knowledge, this is the first report to elucidate the long-term repeated dose effects of CSE on inflammatory exacerbation with organotypic cultured bronchial tissues, and to investigate the transition of inflammatory state in the long term cultivation without any exposures. We believe these findings will contribute to the further use of this *in vitro* model for complex studies.

## 2 Materials and methods

### 2.1 Cell culture

The organotypic cultured bronchial epithelial tissues (MucilAir) and culture medium (MucilAir culture medium) were purchased from Epithelix Sàrl (Geneva, Switzerland). Cell culture was performed in accordance with the manufacturer’s instructions. Separate single donors were used for the four-day exposure study and one-month exposure study; the donors were a 41-year-old Caucasian female and a 28-year old Caucasian male, respectively.

### 2.2 Preparation of the cigarette smoke extract (CSE)

3 R4F reference cigarettes were purchased from the University of Kentucky and conditioned under 22 ± 2 °C and 60 ± 5% relative humidity for at least 48 h before use. Smoke of the 3R4F was generated with a Borgwaldt RM20H smoking machine (Hamburg, Germany) under the Health Canada Intense smoking regimen (a 55-ml puff taken over 2 s, repeated every 30 s) [10]. The total particulate matter was collected on a 45-mm diameter Cambridge filter pad, and then it was extracted with dimethyl sulfoxide purchased from Sigma Aldrich (St. Louis, MO, USA). The initial concentration of total particulate matter was adjusted to 40 mg/ml and appropriately diluted with MucilAir culture medium for subsequent *in vitro* exposures.

### 2.3 Repeated exposure study design

**Fig. 1:**
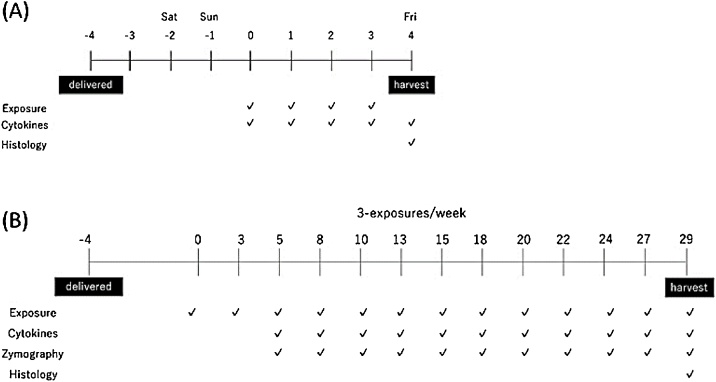
Schematic procedures of repeated CSE exposure studies. (A) Range-finding study design. Cigarette smoke extract (CSE) exposures were performed at 24-h intervals. (B) Repeated-exposure study design. Tissue cultures were exposed to CSE three times per week. Unexposed tissues served as untreated controls.

Two individual repeated CSE exposure studies were conducted: a four-day exposure study with intervals of 24 h and a one-month repeated exposure study with three exposures per week for 29 days. The MucilAir tissues were cultivated at 37 °C in 5% CO_2_ and a humidity of more than 95% beginning at least 96 h prior to the initial CSE exposure. Schematics of the study design are shown in Fig. 1. The tissues were exposed to CSE from the basolateral compartment. Tissues treated with only medium changes were used as non-treatment controls. CSE concentration doses were 5, 10, 20, and 50 μg/ml and 1, 5, 10, and 20 μg/ml CSE for the four-day and one-month exposure study, respectively. The media were collected at each medium change and exposure time and were subjected chemo/cytokine measurement and zymography. The tissue cultures on the day of terminal harvest were subjected to histological evaluation.

### 2.4 Histological analysis

The tissues were fixed in 4% paraformaldehyde (Wako Pure Chemical Industries, Tokyo, Japan) at 4 °C for at least 24 h and then embedded in paraffin. Tissues was sectioned into slices with a 5-μm thickness and stained with hematoxylin and eosin.

### 2.5 Measurement of chemo/cytokines

Concentrations of chemo/cytokines were determined with the Human Cytokine Magnetic Kit (Merck Millipore, Billerica, MA, USA) using the Bio-plex 200 (Bio-Rad, Hercules, CA, USA). Growth factor related oncogene (GRO), interleukin (IL)-8, Interferon gamma-induced protein-10 (IP-10), Monocyte Chemotactic Protein-1 (MCP-1), macrophage inflammatory protein-1β (MIP-1β), regulated on activation, normal T cell expressed and secreted (RANTES), and stromal cell-derived factor 1α (SDF-1α) were measured in the samples obtained from the four-day repeated CSE exposure study. Only GRO and IL-8 were measured in the samples obtained from the one-month repeated CSE exposure study because these chemokines were observed to be highly secreted from the tissues and had shown dose-dependent increases in the preceding four-day CSE exposure study.

### 2.6 Gelatin zymography

The samples obtained from the one-month CSE exposure study were mixed with non-reducing sodium dodecyl sulfate (SDS) sample buffer for polyacrylamide gel electrophoresis. To detect MMP-2 and -9, gelatin zymography was performed. The proteins in the samples were separated with SDS gel electrophoresis in 7.5% acrylamide gels containing 1.0 mg/ml gelatin. After the electrophoresis, the gels were washed twice with 2.5 mM Tris-HCl buffer at pH 7.5 containing 0.5% Triton X-100 and 150 mM NaCl and then incubated in 2.5 mM Tris-HCl buffer at pH 7.5 containing 20 mM NaCl and 10 mM CaCl_2_ for 20 h. The gels were subsequently stained with 0.1% Coomassie blue and photographed using a LAS-4000. The intensities of corresponding bands for each MMP were quantified using Image Quant TL (GE Healthcare, Little Chalfont, UK). The intensity values of the untreated control at each exposure day were used for normalization. Chemicals used for zymography were purchased from Wako Pure Chemical Industries.

### 2.7 Casein zymography

To detect putative MMP-1/3, 0.5 mg/ml casein-containing gels were used. SDS gel electrophoresis was performed at 4 °C, followed by 60-min pre-electrophoresis at room temperature. The remaining experimental procedures were the same as those described above for the gelatin zymography, except for the incubation time for casein zymography, which was 60 h.

### 2.8 Statistical analysis

All presented data are shown as the means and standard errors of triplicate cell culture inserts. Parametric one-way analyses of variance followed by Dunnett’s multiple comparison tests were performed to identify statistically significant differences (set as *p* <  0.05) compared with the untreated control.

## 3 Results and discussion

We first performed a four-day CSE exposure study to determine the suitable dose range for the subsequent one-month repeated CSE exposure study. Schematics of the study designs are shown in Fig. 1A.

**Fig. 2:**
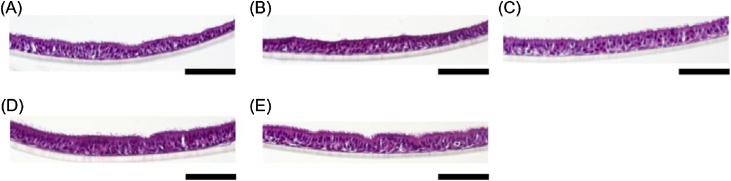
Tissue histology after four CSE exposures (A–E) Representative hematoxylin and eosin staining images of cultures exposed four times to 0 (A), 5 (B), 10 (C), 20 (D), or 50 (E) μg/ml cigarette smoke extract (CSE). The scale bar indicates 100 μm.

Histological evaluation revealed that the tissues exposed to CSE at any of the tested dosages in the four-day exposure study had no obvious morphological alterations, suggesting that these doses were in the low or no toxicity range (Fig. 2).

**Fig. 3:**
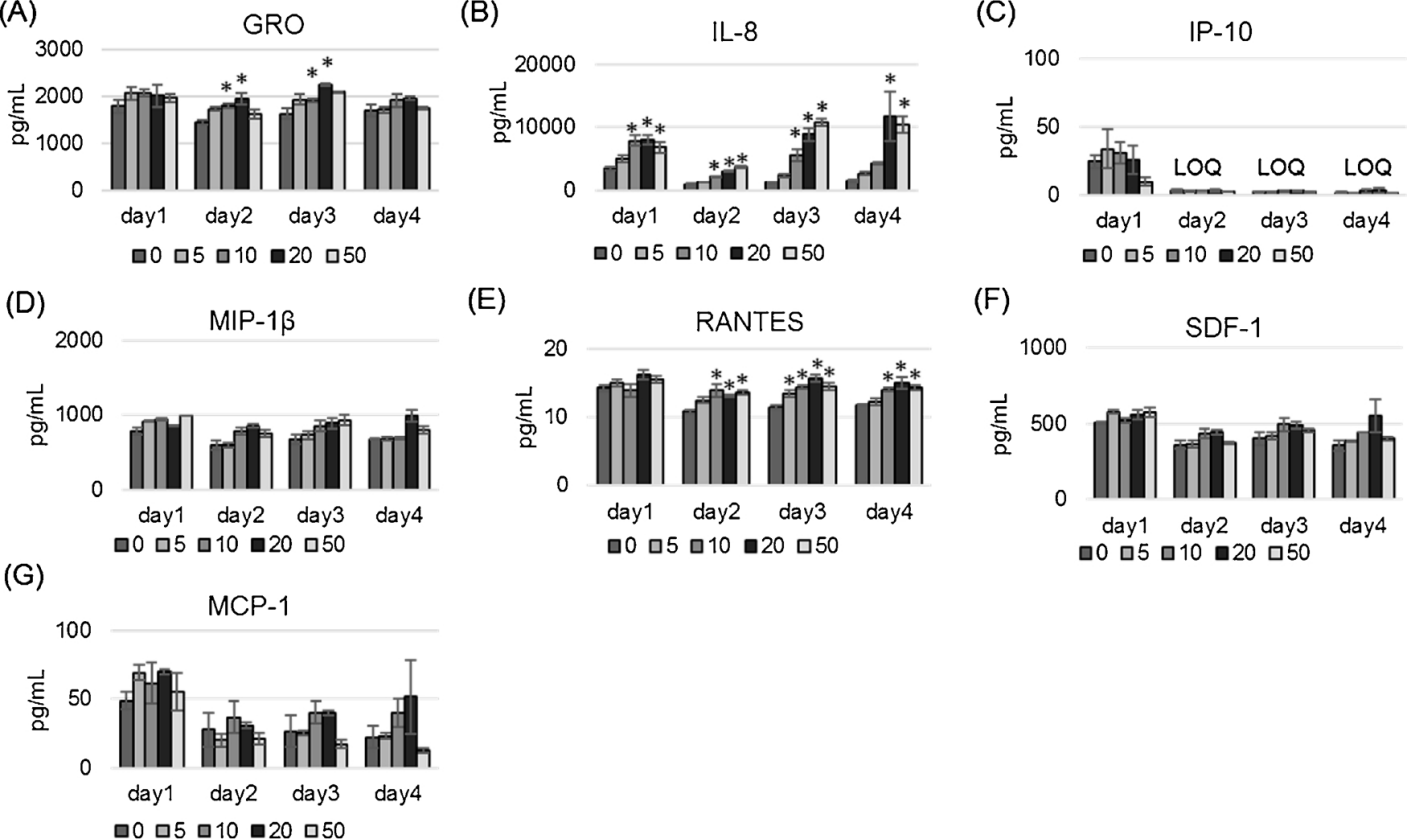
Secretion of seven cyto/chemokines into the basolateral medium. Levels of GRO (A), IL-8 (B), IP-10 (C), MIP-1β (D), RANTES (E), SDF-1 (F), and MCP-1 (G) were measured on days 1–4 after exposure to various doses of CSE (0, 5, 10, 20, or 50 μg/ml). Data are plotted as means of triplicate measurements, and the error bars show standard errors. *Significantly different from untreated controls on each exposure day (*p* <  0.05, one-way ANOVA with Dunnett’s test). LOQ, limit of quantitation.

Additionally, we investigated the cytokine secretion during this short CSE exposure study, and resulted that only IL-8 showed a clear dose response over the experimental period (Fig. 3B), and GRO and RANTES showed a weak dose response and were only partly statistically significant (Fig. 3A and E). Other tested cytokines showed no or weak dose response without statistical significance (Fig. 3C, D, F and G).

It is suggested that the 5–50 μg/ml of CSE is in no or low toxic range, because no apparent toxic effects were observed based on the morphology, but the increased IL-8 levels indicated that inflammation still occurred in the tissues at these doses. Therefore, we decided that the same dose range would be suitable for further extension of the repeated CSE exposure period.

**Fig. 4:**
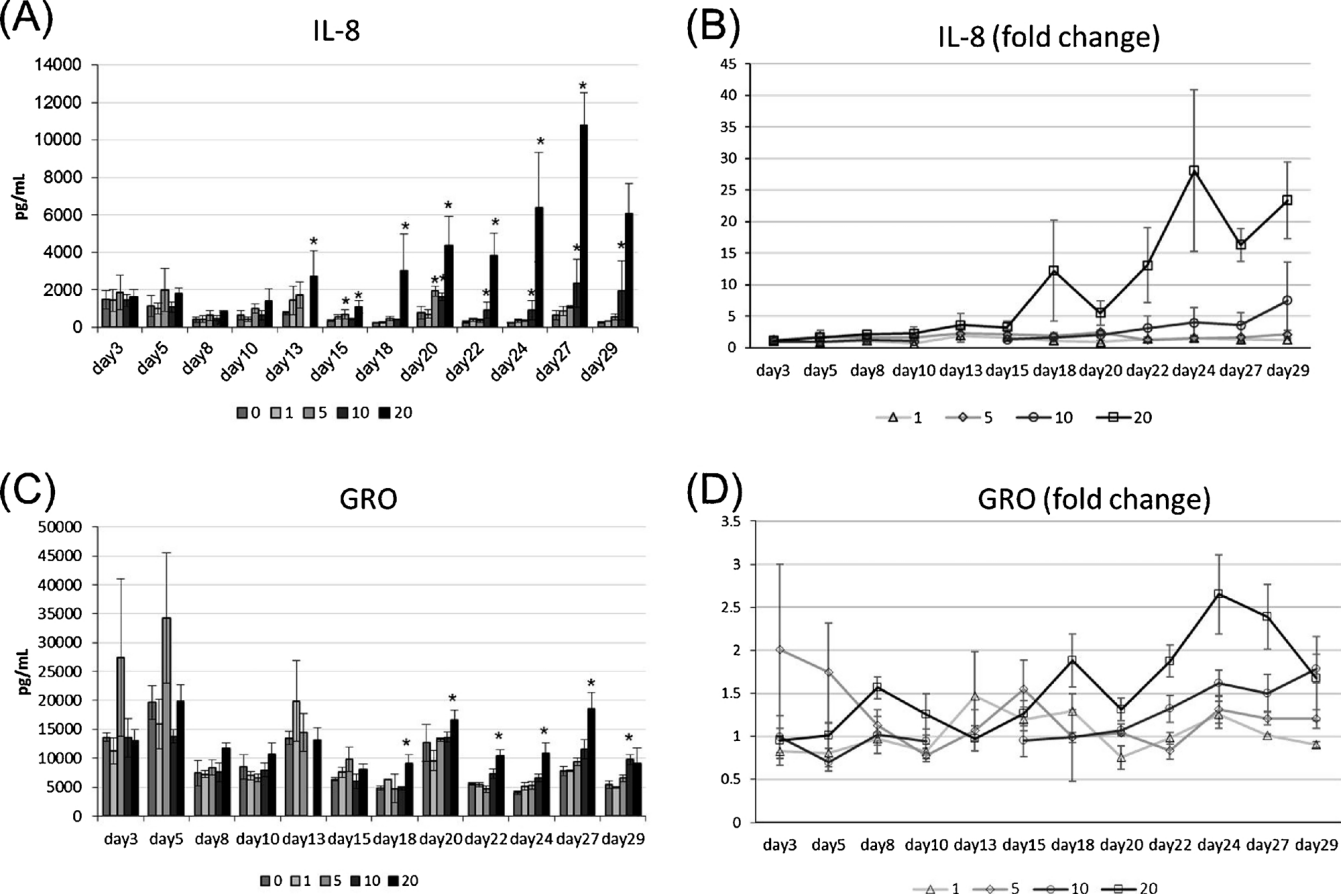
Changes in IL-8 and GRO secretion levels over a one-month period of repeated CSE exposure. Organotypic cultured bronchial tissues were subjected to a one-month period of repeated CSE exposure. (A–B) Quantified IL-8 levels (A) and IL-8 fold increase compared with an untreated control (B) over this period. Data on day 13 at 10 μg/ml is missing due to technical failure. (C–D) Quantified GRO levels (C) and GRO fold increase compared with an untreated control (D) over this period. Data are plotted as means of triplicate measurements, and the error bars show standard errors. *Significantly different from untreated controls on each exposure day (*p* <  0.05, one-way ANOVA with Dunnett’s test).

We next performed a one-month CSE exposure study with three exposures per week, for a total of 13 repeated exposures. Since IL-8 is known to be released from the cells in respiratory tract during both acute and chronic inflammation [11,12], and showed a dose-responsive increase in the short term exposure study (Fig. 3B), we analyzed the secretion of IL-8 to monitor the inflammatory state transition in the one-month exposure study. Additionally, GRO, which is also known to be a biomarker of acute lung injury [13], was measured because as well as IL-8, the secretion level of GRO was significantly higher than the other cytokines. As results, IL-8 levels gradually increased after exposure to more than 10 μg/ml of CSE; no significant induction of IL-8 was observed at day 3, but IL-8 levels finally reached a maximum of 28-fold higher than the non-treatment control at day 24 (Fig. 4A and B). A slight augmentation of GRO levels was found in the case of exposure to 20 μg/ml CSE, and a clear CSE dose dependency of GRO levels was observed after day 18 (Fig. 4C and D), suggesting that the tissues became susceptible to CSE after repeated exposure. Importantly, IL-8 and GRO are considered to act as chemoattractant proteins for neutrophils, which has pivotal roles in the pathogenesis of airway inflammatory diseases by releasing granule proteins, and resulting in further inflammatory exacerbation in respiratory tract [[14], [15], [16]]. Therefore, it is suggested that the augmentation of these cytokines observed in this study could recapitulate the primary responses of cell-specific inflammation that leads to tissue-level inflammatory exacerbation that involves immune cells.

**Fig. 5:**
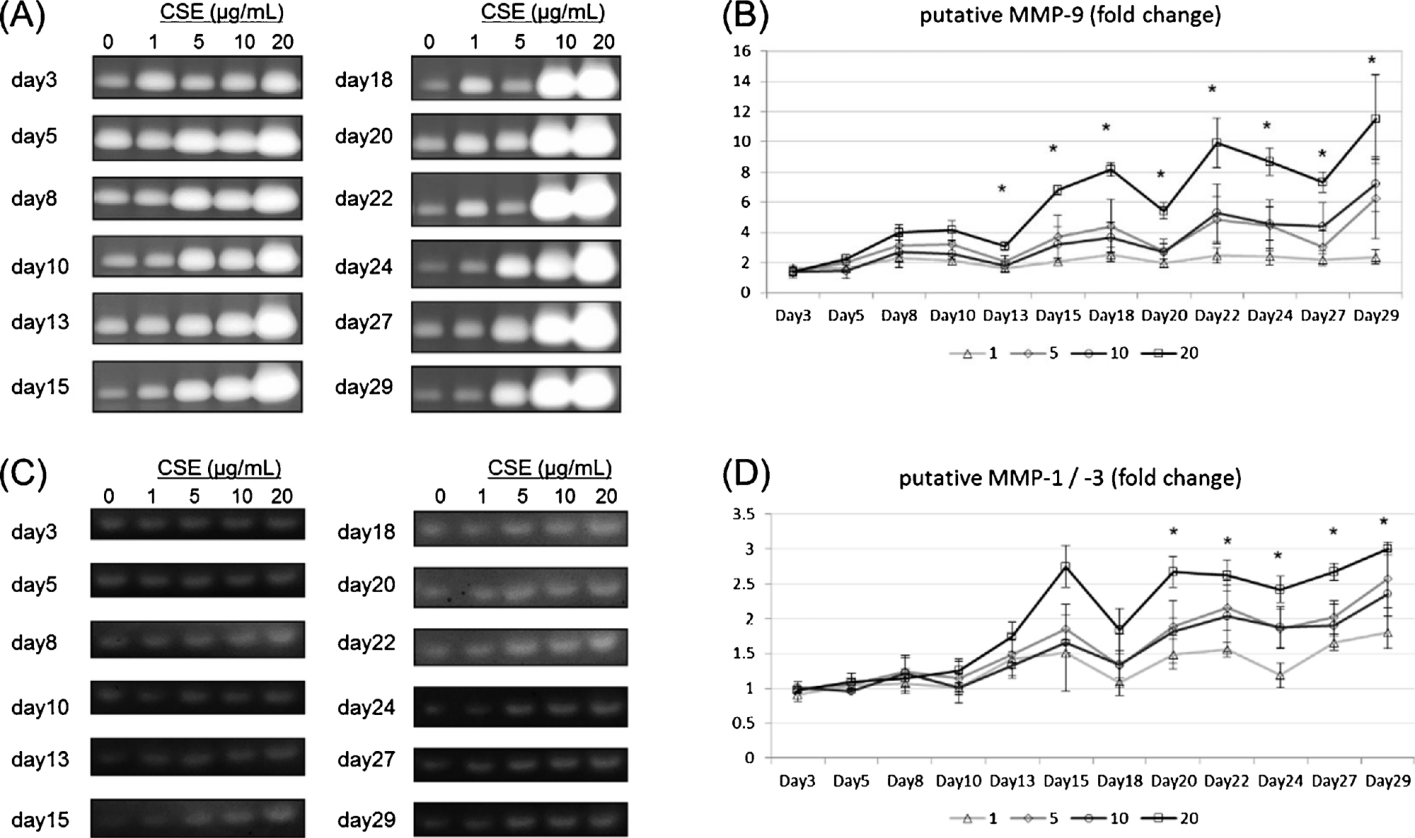
Evaluation of MMP secretion by gelatin and casein zymography. (A) Gelatin zymography was performed to evaluate putative MMP-9 levels. The bands corresponding to pro-MMP-9 are shown. (B) Fold increase of intensity values compared with the untreated control on each exposure day. (C) Casein zymography was performed to evaluate putative MMP-1/3 levels. (D) Fold increase of intensity values compared with the untreated control on each exposure day. Data are plotted as means of triplicate measurements, and the error bars show standard errors. *Significant difference (*p* <  0.05, one-way ANOVA). NT, untreated control.

We also examined MMP secretion because certain kinds of MMPs are known to be related with inflammatory responses [17,18]. We performed gelatin and casein zymographies to evaluate the secreted amounts of MMP-9 and putative MMP-1/3, respectively. The results of both zymographies indicate that the secreted amounts of MMPs increased as the number of exposure increased (Fig. 5A–D).

**Fig. 6:**
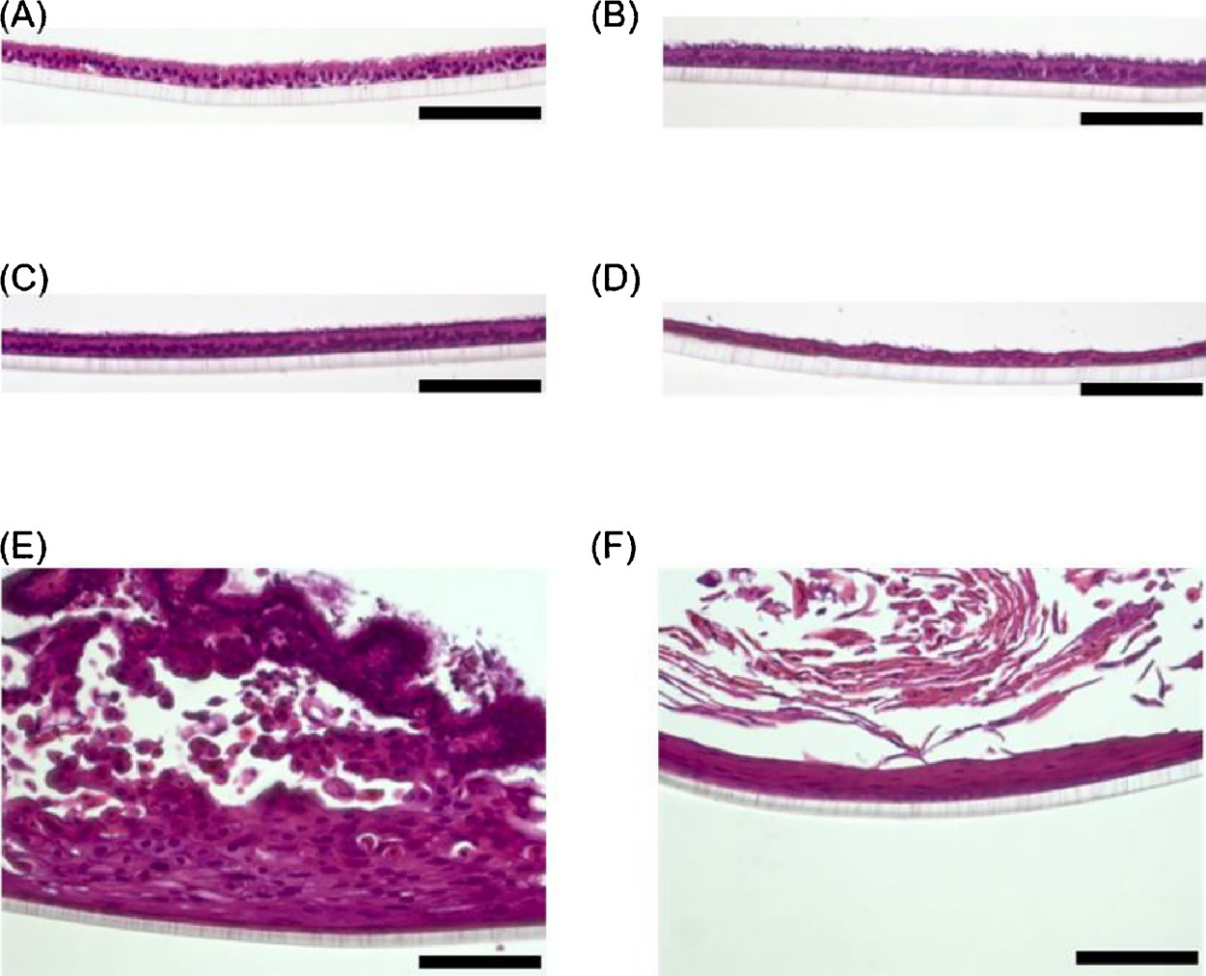
Histology of organotypic cultured bronchial tissues after a one-month period of repeated CSE exposure. Hematoxylin and eosin staining was performed after exposing the organotypic bronchial tissue cultures to each concentration of cigarette smoke extract (CSE) for one month. (A–D) Representative histology images from tissue exposed to 0 (A), 5 (B), 10 (C), or 20 (D) μg/ml CSE. (E–F) Images of abnormal morphology focally observed in tissue exposed to 20 μg/ml CSE. The scale bar indicates 100 μm. Histology images from tissue exposed to 1 μg/ml CSE is not prepared due to technical failure.

Surprisingly, augmentation of MMPs was observed over time, even in the samples exposed to only 1 μg/ml CSE, suggesting that MMPs are more sensitively induced by CSE than the other tested inflammatory cytokines. Normally, MMPs contribute to tissue remodeling [19], and previous work has demonstrated that they are induced by acute injury [20]. Thus, the induction of such MMPs can be elicited by acute effects and may be causative to inducing inflammatory cytokines. Furthermore, our histological analysis also revealed potentially cumulative effects of CSE exposure. In contrast to the results of the four-day CSE exposure study, focal alterations of morphology were observed in the tissues exposed to 20 μg/ml CSE over a one-month period; in these areas, there appeared to be abnormal proliferation and metaplastic alteration (Fig. 6).

Together, these results suggest that repeated CSE exposure elicited cumulative effects on organotypic cultured bronchial epithelial tissues. The present findings are in agreement with our previous report, which demonstrated that pro-inflammatory cytokine secretion is augmented in tissues under chronic, direct exposure to whole CS [9]. The test substance used here is a CS extract, which contains other chemicals than the ones present in the gas and vapor phases; therefore, inflammatory exacerbation may be attributable to such chemicals.

**Fig. 7:**
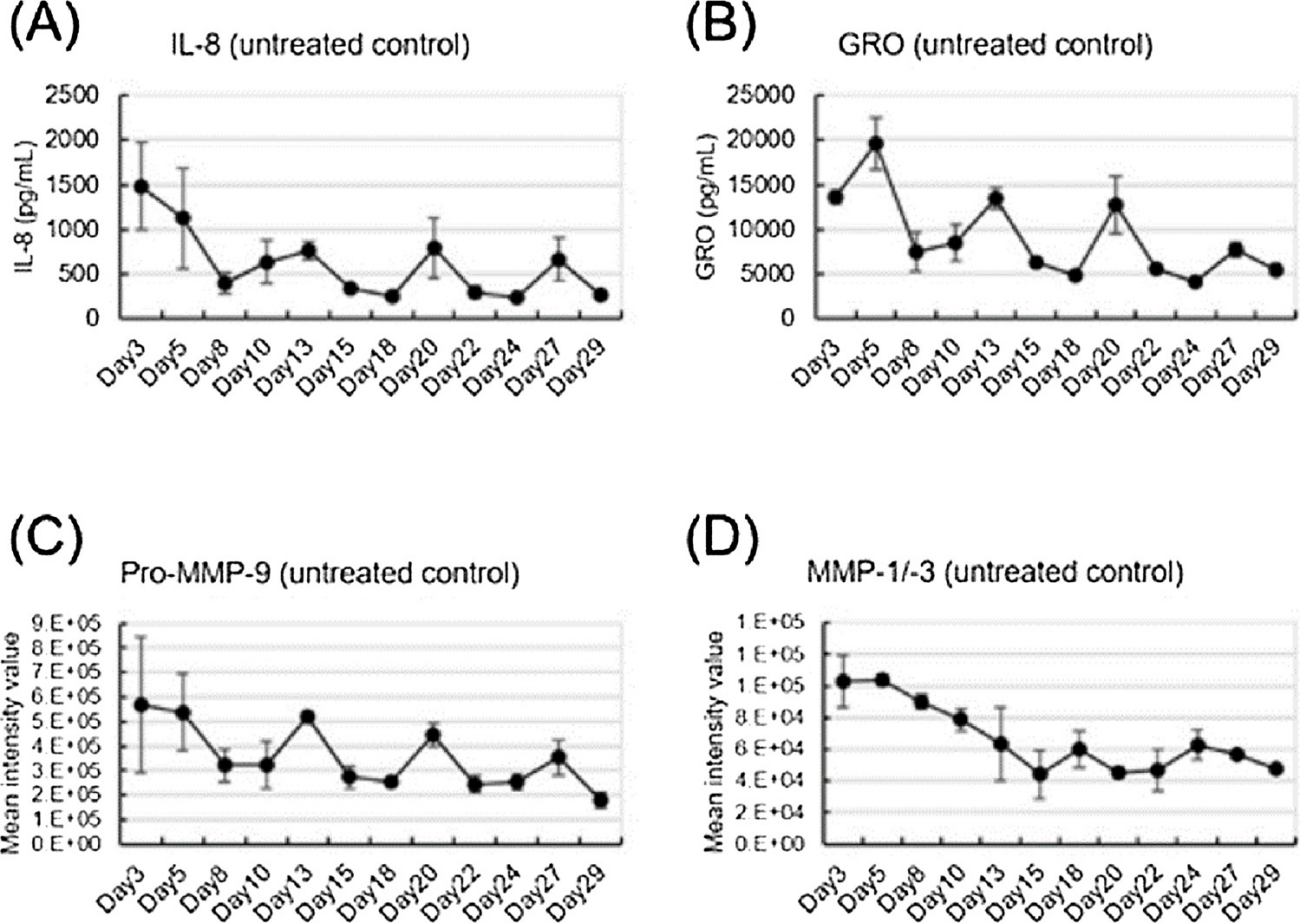
Spontaneous secretion of inflammatory mediators. The secretion of inflammatory mediators IL-8 (A), GRO (B), MMP-9 (C), and MMP-1/-3 (D) from untreated control tissues on each CSE exposure day.

We additionally investigated the changes from the baseline levels of cytokines and MMPs in non-treated controls during the one-month CSE exposure experimental period. These measurements revealed that the secretions of inflammatory mediators from the non-treated controls were relatively high at the start of the study but declined by day 8 (Fig. 7A–D). Based on this finding, it is possible that although the organotypic cultured bronchial epithelial tissues are shipped in a fully differentiated state, they may become damaged during shipping. Furthermore, after day 8, periodic increases and decreases were found in the secretions of IL-8, GRO, and MMP-9, and these changes seemed to be synchronized (Fig. 7A–C).

Thus, there may be some non-24 h circadian-like rhythm in the tissues that can show spontaneous inflammatory responses. These results are noteworthy because they suggest that the timing of exposure is likely critical for the interpretation of study results, especially for single-exposure studies. Additionally, to avoid confounding the data, we propose that organotypic cultured bronchial epithelial tissues be subjected to an acclimation period during which the tissues are cultured with only medium changes until their inflammatory responses triggered by shipping calm down.

## 4 Conclusions

The results of these two repeated CSE exposure studies indicate that the organotypic cultured bronchial tissues show cumulative effects from repeated exposure to CSE doses with low or no toxicity and are therefore a useful tool for the evaluation of tissue-specific sub-chronic effects. In addition, our findings also support the implementation of an acclimation period for organotypic cultured bronchial tissues before use to avoid potentially confounding factors.

## Conflict of interest

The authors are employees of Japan Tobacco Inc. and declare that they have no conflicts of interest.

## Funding information

This research was sponsored by Japan Tobacco Inc.
